# Intestinal parasitic infections and associated factors among people living with HIV attending Dessie Referral Hospital, Dessie town, North-east Ethiopia: a cross-sectional study

**DOI:** 10.1186/s12981-022-00443-6

**Published:** 2022-04-20

**Authors:** Daniel Getacher Feleke, Abdurahaman Ali, Habtye Bisetegn, Mengaye Andualem

**Affiliations:** 1 Department of Microbiology, Immunology and Parasitology, College Health Sciences, Addis Ababa University, Addis Ababa, Ethiopia; 2 Department of Medical Laboratory Science, College of Medicine and Health Sciences, Wollo University, Dessie, Ethiopia

**Keywords:** Dessie, HIV/AIDS, ART, Intestinal parasites, Ethiopia

## Abstract

**Introduction:**

Intestinal parasites and HIV/AIDS co-infection become a major public health concern in Africa. The management and care of HIV/AIDS patients is being complicated by intestinal parasitic infections. Therefore, this study aimed to determine the prevalence and associated factors of intestinal parasitic infections among people living with HIV at Dessie Referral Hospital, North-east Ethiopia.

**Methods:**

This cross sectional study was conducted from March to May 2019. Systematic simple random sampling technique was used to recruit study participants. Stool specimen was collected and examined microscopically using wet mount, formol-ether concentration technique and modified Zeihl–Neelsen methods. Socio-demographic characteristics and associated factors were collected using structured questionnaire. The recent CD4 cell count was obtained from patients ART follow-up record. Data were analysed using SPSS version 20 software. Bivariate and multivariate logistic regression was done to investigate the association between independent and dependent variables**.**

**Results:**

Of the total of 223 study participants 120 (53.8%) were females and 162 (72.6%) were urban resident. The overall prevalence of intestinal parasites was 47 (21.1%). Eleven different intestinal parasites species were detected. The dominant intestinal parasite species was *Entameoba* *histolytica* 14 (6.3%) followed by *Enterobius vermicularis* 5 (2.2%). Multivariate logistic regression analysis showed that individuals who had a habit of hand washing after latrine were less likely to be infected with intestinal parasitic infection (AOR 0.15, 95% CI 0.05–0.412). On the other hand individuals who had CD_4_ cell count of < 200 cells/ml^3^ were 45.53 times more likely infected with intestinal parasites.

**Conclusion:**

The prevalence of intestinal parasite was higher than previous report from the same study area almost a decade ago. There was statistical significant association between hand washing habit after latrine, habit of eating raw vegetables and CD_4_ cell count less than 200 cells/ml^3^ and intestinal parasitic infections. Health education program interrupted in Dessie referral hospital should be continued to reduce the prevalence of intestinal parasites. Utilization of water treatment, washing hand after latrine and eating cooked or appropriately washed vegetables should also be promoted. Moreover, periodic laboratory stool specimen examination and prompt treatment are necessary.

## Background

Intestinal parasitic infections are widely distributed throughout the world and they are a major cause of morbidity and mortality [1]. They are usually highly prevalent in sub-Saharan Africa, where HIV/AIDS cases are also concentrated [2]. The co-infection of intestinal parasites and HIV/AIDS has become a major public health concern in Africa. Human immune deficiency (HIV) infection suppress human immunity and expose individuals to opportunistic infections is responsible for high mortality rate [3]. Among the opportunistic infections, intestinal parasitic infections caused by helminths and protozoa are the most common infections in the world [4]. Intestinal parasitic infections become severe when they appear in HIV/AIDS patients. They play an important role in the progression of HIV infection to AIDS, by further disturbing the immune system [1]. The common immunopathogenetic basis for the deleterious effects that parasitic diseases may involve in preferential activation of the T helper type process. Thus combating the HIV should involve control and prevention of parasitic diseases [5].

The prevalence of both HIV/AIDS and intestinal parasites in Ethiopia is high. In Ethiopia the national adult HIV prevalence is 0.9% and an estimated 613,000 people were living with HIV, and 30% were from the Amhara region in 2017 [6]. The incidence rate of HIV in Dessie was higher than other towns of Amhara region with 5.74 per 1000 population [6]. The high occurrence of associated factors such as low coverage of insufficient water supply for drinking, low coverage of self and environmental hygiene facilities and contamination of food and drinking water that results from improper disposal of human excreta favour the high prevalence of intestinal parasitic infections [7, 8]. In Ethiopia, intestinal parasitic infections are responsible for more than half a million annual visits of the outpatient services of the health institutions [9]. This report may be underestimated due to lack of appropriate diagnostic test methods in many of the health institutions. Intestinal parasites and HIV/AIDS are widespread throughout the country [8, 9]. The management and care of HIV/AIDS patients is complicated by intestinal parasitic infections. So, detecting intestinal parasites and understanding the health complications they cause will help health workers to manage properly and treat HIV/AIDS patients. As far as we know, there was only one study conducted 9 years ago among pre-ART and on ART HIV/AIDs patient in the study area. Therefore, this study was aimed to update the information about the prevalence and associated factors of intestinal parasitic infection among people living with HIV who are on ART follow up at Dessie Referral Hospital, Dessie town, North-East Ethiopia.

## Methods

### Study area

This study was conducted at Dessie Referral Hospital which is found in Dessie town, North-east Ethiopia. Dessie town is found in western part of Amhara region and it is 401 km far from Addis Ababa, the capital city of Ethiopia. It is geographically located at 11°8N latitude, 39°38E longitude, and an average altitude of 2470 m above sea level. The town is generally characterized by cold weather with mean annual maximum and minimum temperature of 25 °C and 5 °C, respectively. Based on the 2007 census conducted by the Central Statistical Agency of Ethiopia, Dessie town has a total population of 151,174 of whom 72,932 are men and 78,242 women. A study in Amhara region reported that incidence rate of HIV in Dessie was higher than other towns of Amhara region with 5.74 per 1000 population [6].

### Study design and period

This institution based cross sectional study was conducted from March to May 2019.

### Inclusion and exclusion criteria

All HIV/AIDS patients visited ART clinic of Dessie Referral Hospital during the study period were included. Individuals who were on intestinal parasitic infection treatment during the data collection period were excluded.

### Sample size and sampling technique

The sample size was determined using the single population proportion formula using a prevalence of 17.6% [8] with margin of error 5% and 95% confidence interval [Z (1− ά/2) = 1.96]. The final calculated sample size was 223 and systematic random sampling technique was used to recruit the study participants using the ART clinic registration book as a sampling frame.

### Data collection

Socio-demographic and associated factors were collected using pre-tested structured questionnaire. Data collectors were trained before the start of data collection. Information about recent CD4 cell count of people living with HIV was obtained from their ART follow-up record. An estimated 5 g stool specimen was collected from each study participant using leak proof and clean plastic container for parasitological examination.

### Laboratory investigation

Stool sample was examined microscopically using direct wet mount, Formol–ether concentration technique and modified Ziehl–Neelsen method for parasite detection. Direct wet mount examination was performed using normal saline and Lugol’s iodine at Dessie referral Hospital. Then the remained stool sample was preserved with 10% formalin for formol–ether concentration technique and modified Zeihl–Neelsen method procedures. For formol-ether concentration technique, an estimated 1 g (pea-size) stool sample was mixed with 4 ml of 10% formol water. Additional 4 ml of 10% v/v formol water was added and mixed by shaking. After the content sieved 4 ml of diethyl ether was added and mixed for 1 min. Then it was immediately centrifuged at 3000 revolution per minute (rpm) for 1 min. Finally, the sediment was examined under microscope. The remaining sediment was stained using Modified Ziehl–Neelsen method for the detection of intestinal coccidian parasites. Standard Operational Procedure (SOP) was strictly followed during microscopic examination of stool specimen for intestinal parasites.

### Data analysis

Data were checked for completeness and were entered to SPSS version 20 software and analysed. Logistic regression were done to investigate the association between independent variables and dependent variable. In all comparisons, p-value less than 0.05 was considered statistically significant.

### Ethical consideration

The willingness of the study participants were asked using written consent form. Ethical clearance was obtained from Wollo University, College of Medicine and Health Science, Department of Medical Laboratory Science. Study participants with positive intestinal parasite result were treated in Dessie Referral Hospital according to the national treatment guideline.

## Result

### Socio-demographic characteristics and associated factors

Of the total of 223 study participants 120 (53.8%) were females, 108 (48.4%) were between 30 and 49 age group, 162 (72.6%) were urban resident, 100 (44.8%) were single and majority of them 92 (41.3%) were at primary educational level. Majority (84.3%) of the study participants had the habit of using latrine. With regard to water source for drinking, 82.5% of study participants use pipe water. Almost all of the study participants 216 (96.9%) had no the habit of using water treatment (Table 1).

**Table 1:** Socio-demographic characteristics and associated factors among people living with HIV attending ART clinic of Dessie Referral Hospital, North-East Ethiopia from March to May 2019

Variables	Category	N (%)
Sex	Male	103 (46.2)
	Female	120 (53.8)
Age	10–29	74 (33.2)
	30–49	108 (48.4)
	> 50	41 (18.4)
Marital status	Single	100 (44.8)
	Married	89 (39.9)
	Divorced	27 (12.1)
	Widowed	7 (3.1)
Educational status	Illiterate	60 (26.9)
	Primary educational level	92 (41.3)
	Secondary educational level	56 (25.1)
	College and above	15 (6.7)
Residence	Urban	162 (72.6)
	Rural	61 (27.4)
Availability and usage of latrine	Always	188 (84.3)
	Sometimes	29 (13)
	Never	6 (2.7)
Hand washing after latrine	Always	149 (66.8)
	Some times	74 (33.2)
Eating raw vegetable	Yes	109 (48.9)
	No	114 (51.1)
Eating raw meat	Yes	48 (21.5)
	No	175 (78.5)
Source of water drinking	Well water	6 (2.7)
	Tap water	184 (82.5)
	Spring water	25 (11.2)
	River water	8 (3.6)
Utilization of water treatment	Yes	7 (3.1)
	No	216 (96.9)
Shoes wearing	Always	197 (88.3)
	Some time	26 (11.7)
Finger nail status	Trimmed	217 (97.3)
	Not trimmed	6 (2.7)
CD_4_ count	< 200 CD_4_ cell/ml^3^	15 (6.7)
	201–499 CD_4_ cell/ml^3^	111 (49.8)
	> 500 CD_4_ cell/ml^3^	97 (43.5)

### Prevalence of intestinal parasites among people living with HIV

**Fig. 1:**
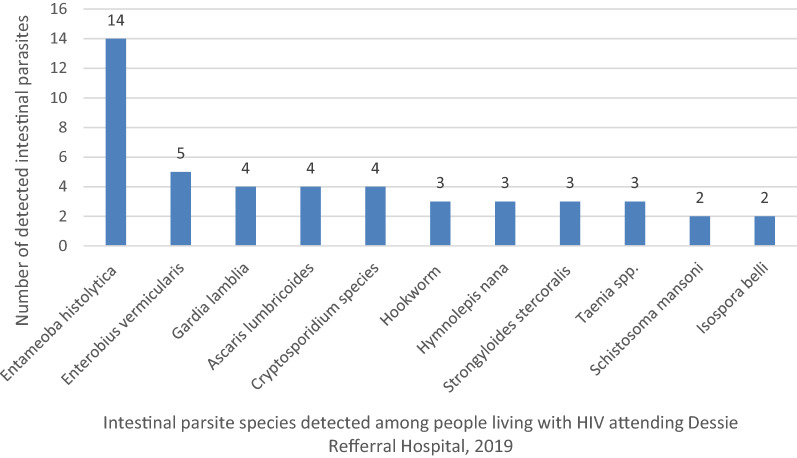
intestinal parasites detected among individuals living with HIV attending ART clinic of Dessie Referral Hospital from March to May 2019.

The overall prevalence of intestinal parasites was 47 (21.1%). Eleven different intestinal parasites species were detected. The dominant intestinal parasite species was *Entameoba histolytica* 14 (6.3%) followed by *Enterobius vermicularis* 5 (2.2%), *Gardia lamblia* 4 (1.8%), *Ascaris lumbricoides* 4 (1.8%) and *Cryptosporidium* species 4 (1.8%). The least common parasites detected in this study were *Isospora belli* and *Schistosoma mansoni* (Fig. 1).

### Associated factors for intestinal parasites among people living with HIV

Bivariate analysis showed that hand washing habit after latrine, habit of eating raw vegetables, eating raw meat and CD4 count showed statistical significant association with intestinal parasitic infection. In multivariate logistic regression analysis only hand washing habit after latrine, CD4 cell count of less than 200cells/ml^3^ and habit of eating raw vegetables had statistical significant association with intestinal parasitic infection. There was statistical significant association between intestinal parasitic infections and hand washing habit. Those individuals who had hand washing habit after latrine were less likely to be infected with intestinal parasites (AOR 0.15, 95% CI 0.05–0.412). Similarly, individuals who had CD4 cell count of < 200 cells/ml^3^ were 45.53 times more likely infected with intestinal parasites compared to those who had > 500 CD_4_ cells/ml^3^ (Table 2).

**Table 2:** The association of intestinal parasitic infection and risk factors among people living with HIV attending ART clinic of Dessie Referral Hospital from March to May 2019

Variables	Categories	Intestinal parasitic infection		P-value	COR (95%CI)	P-value	AOR (95% C.I)
		Positive N (%)	Negative N (%)				
Hand washing after latrine	Always	12 (29.3)	137 (75.3)	0.00	0.136 (0.06–0.29)	<0.01*	0.15 (0.05–0.41)
	Sometimes	29 (70.7)	45 (24.7)	1		1	
Eating raw vegetable	Yes	38 (92.7)	71 (39.0)	0.00	19.8 (5.89–66.58)	<0.01*	13.66 (3.52–53.06)
	No	3 (7.3)	111 (61)	1		1	
Eating raw meat	Yes	19 (46.3)	31 (17%)	0.00	4.21 (2.04–8.69)	0.079	2.35 (0.91–6.09)
	No	22 (53.7)	151 (83)	1		1	
CD4 cell count	< 200 CD4 cell/ml^3^	10 (24.4)	5 (2.7%)	0.00	36.8 (9.07–149.36)	< 0.01*	45.53 (7.46–277.70)
	200-499 CD4 cell/ml^3^	26 (63.4)	85 (46.7)	0.001	5.63 (2.07–15.32)	0.012*	4.38 (1.38–13.87)
	> 500 CD4 cell/ml^3^	5 (12.2)	92 (50.5)	1		1	

^*^Statistically significant

*COR* crude odds ratio, *AOR* adjusted odds ratio

## Discussion

Intestinal parasites and HIV/AIDS are co-endemic in many regions of the world [10]. In this study, majority of the study participants (53.8%) were females. This was in agreement with a report from Kano, Nigeria [11]. The prevalence of HIV/AIDS may be relatively increased in women as receptive partner in heterosexual intercourse [11]. In the present study majority of the HIV/AIDS patients were single (44.8%). This could be one of the possible reasons for being exposed to HIV/AIDS as a result of having multiple sexual partner.

This study revealed that the prevalence of intestinal parasites among people living with HIV was 47 (21.1%). The finding was higher than previous studies reported from Ethiopia (13.9%) [12], Nigeria (11.4%) [11] and Cameroon (14.64%) [12, 13]. On the other hand the prevalence was lower than reports from Cameroon (57.48%) and Ethiopia [3, 14, 15]. The lower or higher prevalence of intestinal parasites in this study compared to previous studies might be due to variations in geographical area that affect the distribution of parasites, study period (seasonal variations), life style and culture of the society, awareness about the prevention and control of intestinal parasites, differences in socio economic status and the diagnostic methods employed for parasite detection.

The prevalence of intestinal parasites was also higher than the study conducted in Dessie Referral Hospital 9 years ago among HIV/AIDS patients (17.6%) [8]. The possible reasons might be due to the interruption of scheduled deworming program to people taking ART at the study site. The other most probable reason could be the interruption of health education programs that was given for HIV/AIDS patients at Dessie Referral Hospital about the transmission, prevention and control of intestinal parasites and other infectious diseases.

With regard to the species of intestinal parasites, the most common parasite detected in this study was *E. histolytica* (6.3%) and *E. vermicularis* (2.2%). The prevalence of *E. histolytica* was higher than a study conducted among HIV/AIDs patients reported from Benin City, Nigeria (3.3%) [2]. On the other hand it was consistent with studies conducted in Cameroon (7.52%) [12] and Lesotho (6.7%) [16].

In the present study there was statistically significant association between CD4 cell count and intestinal parasitic infection among HIV/AIDs patients. This was consistent with previous studies [15, 17, 18]. Similarly, hand washing habit after toilet and eating raw vegetables showed statistical significant association with intestinal parasitic infection. These factors are known to expose individuals for intestinal parasitic infections. Especially, eating raw vegetables that are not washed appropriately before eating is the major source of intestinal parasitic infections. The present study has limitations with regard to Microsoporidia, *Strongyloides stercoralis* and *Enetrobius vermicularis* diagnosis. Although the diagnosis of these parasites require specific and sensitive diagnostic methods, we could not employ special diagnostic techniques due to lack of resources. Therefore, the prevalence these parasites might be underestimated in this study.

## Conclusion

The prevalence of intestinal parasite was higher than previous report from the same study area almost a decade ago. The dominant intestinal parasites detected were *E. histolytica* followed by *E. vermicularis*. The present study also revealed statistical significant association between hand washing habit after latrine, eating raw vegetables and CD_4_ cell count and intestinal parasitic infections. The interrupted health education program in the hospital should be continued to reduce the prevalence of intestinal parasites. Utilization of water treatment, washing hand after latrine and eating cooked or appropriately washed vegetables should also be promoted. In addition, periodic laboratory stool specimen examination, awareness creations about the prevention and control of intestinal parasites and prompt treatment are necessary.

## Data Availability

The authors confirm that all data underlying the findings are fully available without restriction. All relevant data are within the manuscript.

## Abbreviations

AIDS: Acquired immunodeficiency syndrome
ART: Anti-retroviral therapy
CD4: Cluster of differentiation on T_4_ lymphocyte cells
HIV: Human immunodeficiency virus
